# Self-Assembly of Decoupled Borazines on Metal Surfaces: The Role of the Peripheral Groups

**DOI:** 10.1002/chem.201402839

**Published:** 2014-07-30

**Authors:** Nataliya Kalashnyk, Praveen Ganesh Nagaswaran, Simon Kervyn, Massimo Riello, Ben Moreton, Tim S Jones, Alessandro De Vita, Davide Bonifazi, Giovanni Costantini

**Affiliations:** a Department of Chemistry, University of Warwick Gibbet Hill Road, Coventry, CV4 7AL (UK); b Physics Department, King's College London London, WC2R 2LS (UK); c Namur Research College (NARC) and Department of Chemistry, University of Namur (UNamur) Rue de Bruxelles 61, 5000 Namur (Belgium); d Department of Pharmaceutical and Chemical Sciences and INSTM UdR Trieste, University of Trieste Piazzale Europa 1, 34127 Trieste (Italy)

**Keywords:** borazines, molecular dynamics, scanning tunnelling microscopy, self-assembly, surface chemistry

## Abstract

Two borazine derivatives have been synthesised to investigate their self-assembly behaviour on Au(111) and Cu(111) surfaces by scanning tunnelling microscopy (STM) and theoretical simulations. Both borazines form extended 2D networks upon adsorption on both substrates at room temperature. Whereas the more compact triphenyl borazine **1** arranges into close-packed ordered molecular islands with an extremely low density of defects on both substrates, the tris(phenyl-4-phenylethynyl) derivative **2** assembles into porous molecular networks due to its longer lateral substituents. For both species, the steric hindrance between the phenyl and mesityl substituents results in an effective decoupling of the central borazine core from the surface. For borazine **1**, this is enough to weaken the molecule–substrate interaction, so that the assemblies are only driven by attractive van der Waals intermolecular forces. For the longer and more flexible borazine **2**, a stronger molecule–substrate interaction becomes possible through its peripheral substituents on the more reactive copper surface.

## Introduction

The rapid development of molecule-based technologies that has started to permeate our everyday life has created a new impetus for studying the interaction and supramolecular assemblies of functional molecular units on surfaces as these lie at the heart of several device architectures.^1^–^3^ Deposition of molecules on conducting electrodes may significantly affect their intrinsic chemical, optic and magnetic properties that can result in a correspondently significant modification of the structural and electronic characteristics of the molecule.^4^

Such strong coupling is not always desirable. Indeed, it is often necessary to pursue an electronic decoupling of the molecules from the conductive substrate as, for example, is frequently done for organic adsorbates used in molecular electronics.^5^ Among the different approaches, the most widely used method consists of sandwiching ultrathin insulating films, for example, organic layers,^6^ inorganic salts^4^, ^7^ or oxides,^8^, ^9^ between the metallic substrate and the molecular layer. An alternative approach is to exploit molecular systems bearing peripheral bulky alkyl groups that preserve their electronic properties by lifting their functional cores from the substrate.^10^–^12^ Recent examples include derivatives of azobenzene,^13^, ^14^ oligophenylene-ethynylene,^15^ porphyrin,^16^–^18^ corannulene^19^ and antracene,^20^, ^21^ all adsorbed on metallic surfaces.

Functional polycyclic aromatic hydrocarbons (PAHs) are among the most promising active molecular materials for optoelectronics.^22^–^25^ Among the different functionalisation approaches, the replacement of carbon atoms by isostructural elements has emerged as a versatile strategy to tune their optoelectronic and mechanical characteristics.^26^ In particular, the replacement of C=C bonds by covalent B—N couples leads to molecular isosteres displaying a strong local dipole moment^27^–^31^ responsible for their peculiar electronic and self-assembly properties.^32^–^35^ Specifically, borazine derivatives^36^–^42^ have been used to fabricate materials for a large variety of optoelectronic devices.^43^–^55^

**Scheme 1:**
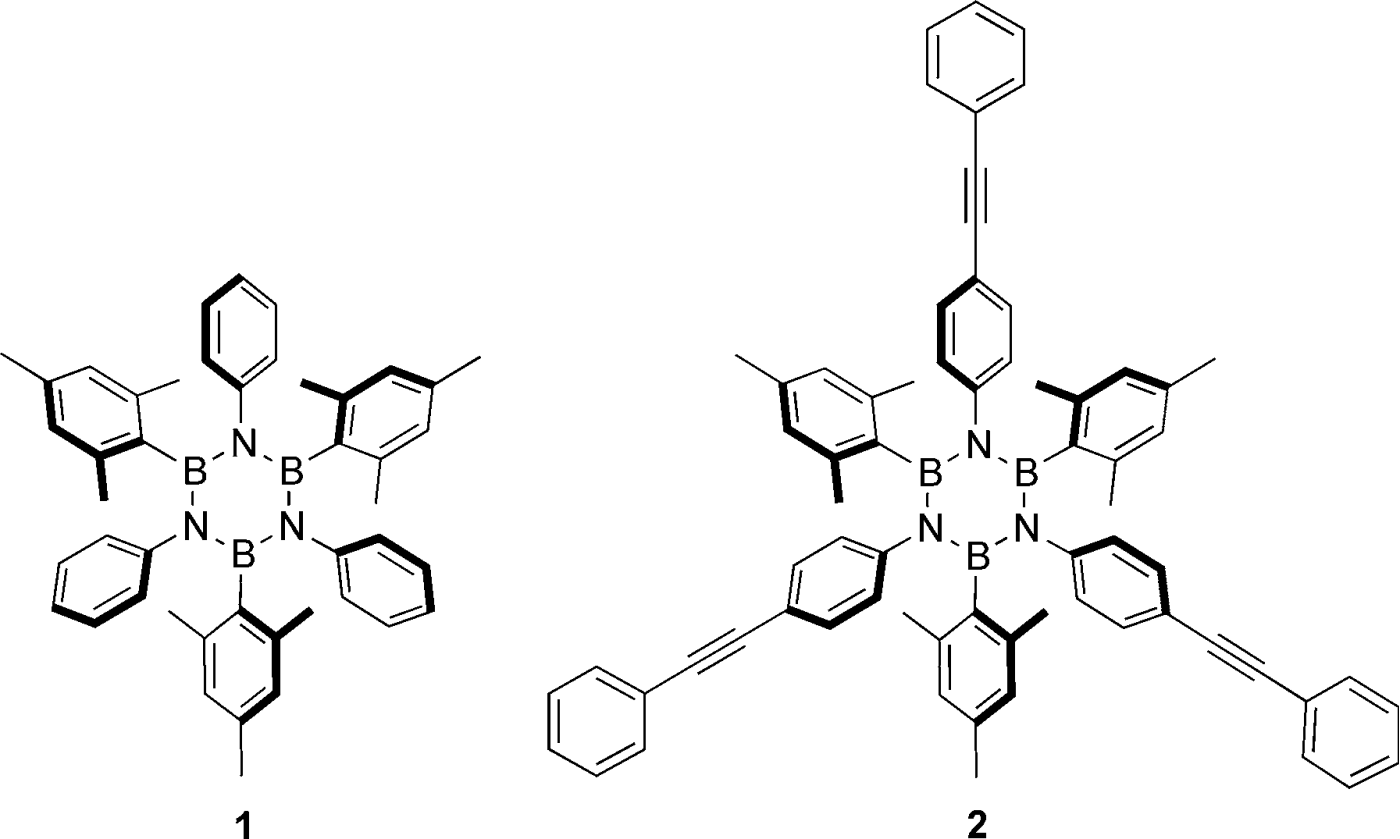
Chemical structures of borazines 1 and 2.^56^, ^57^

In this respect, we have recently showed that B-trimesityl-*N*-triphenylborazine (molecule **1**, Scheme 1) can be processed as an active layer for engineering light-emitting electrochemical cell (LEC) devices operating in the UV region.^56^ In another endeavour, we also reported the first self-assembly behaviour of borazines adsorbed on Cu(111) surfaces. Ruled by a delicate interplay of short-range van der Waals (vdW) attractions and long-range Coulomb repulsions between molecules, we have shown that a hydroxyl-pentaaryl borazine assembles in small clusters, which is in striking contrast with the large-island assembly obtained for molecule **1** (Scheme 1).^57^

In the present work we turn to investigating how the self-assembly of two borazine derivatives (molecules **1** and **2**, Scheme 1) is affected by the presence of lateral groups which, while driving the assembly, can at the same time interact with the metal substrate.

Specifically, we carried out a ultra-high vacuum scanning tunnelling microscopy (UHV-STM) study of the self-assembly of molecules **1** and **2** on Au(111) and Cu(111). For the reference borazine **1**, carrying only short lateral groups, this produced the same close-packed supramolecular architectures on both substrates. Borazine **2** was instead found to yield different networks on the two surfaces. We rationalised this behaviour with the help of molecular dynamics (MD) simulations suggesting that a very effective decoupling of the central borazine core from the substrate occurs for both molecules, whereas a pronounced molecule–substrate interaction through its longer peripheral substituents is predicted for borazine **2** only on the more reactive Cu(111) surface.

## Results and Discussion

### Synthesis of the borazine molecular modules

The molecular structures of *N*-triphenyl-B-trimesityl (**1**) and *N*-tri(phenyl-4-phenylethynyl)-B-trimesityl (**2**) borazine molecules are shown in Scheme 1. Whereas compound **1** features three phenyl (Ph) and three mesityl (Mes) groups bonded to the nitrogen and boron atoms, respectively, molecule **2** replaces the phenyl groups with peripherally protruding phenyl-4-ethynylphenyl moieties. Following the experimental protocols developed by us for borazine **1**,^56^, ^57^ molecule **2** was obtained after reaction of 4-ethynylphenylaniline with BCl_3_ upon subsequent addition of three equivalents of MesLi following the route displayed in the Supporting Information.

### Self-assembly of molecule 1 on Cu(111) and Au(111) surfaces

**Figure 1:**
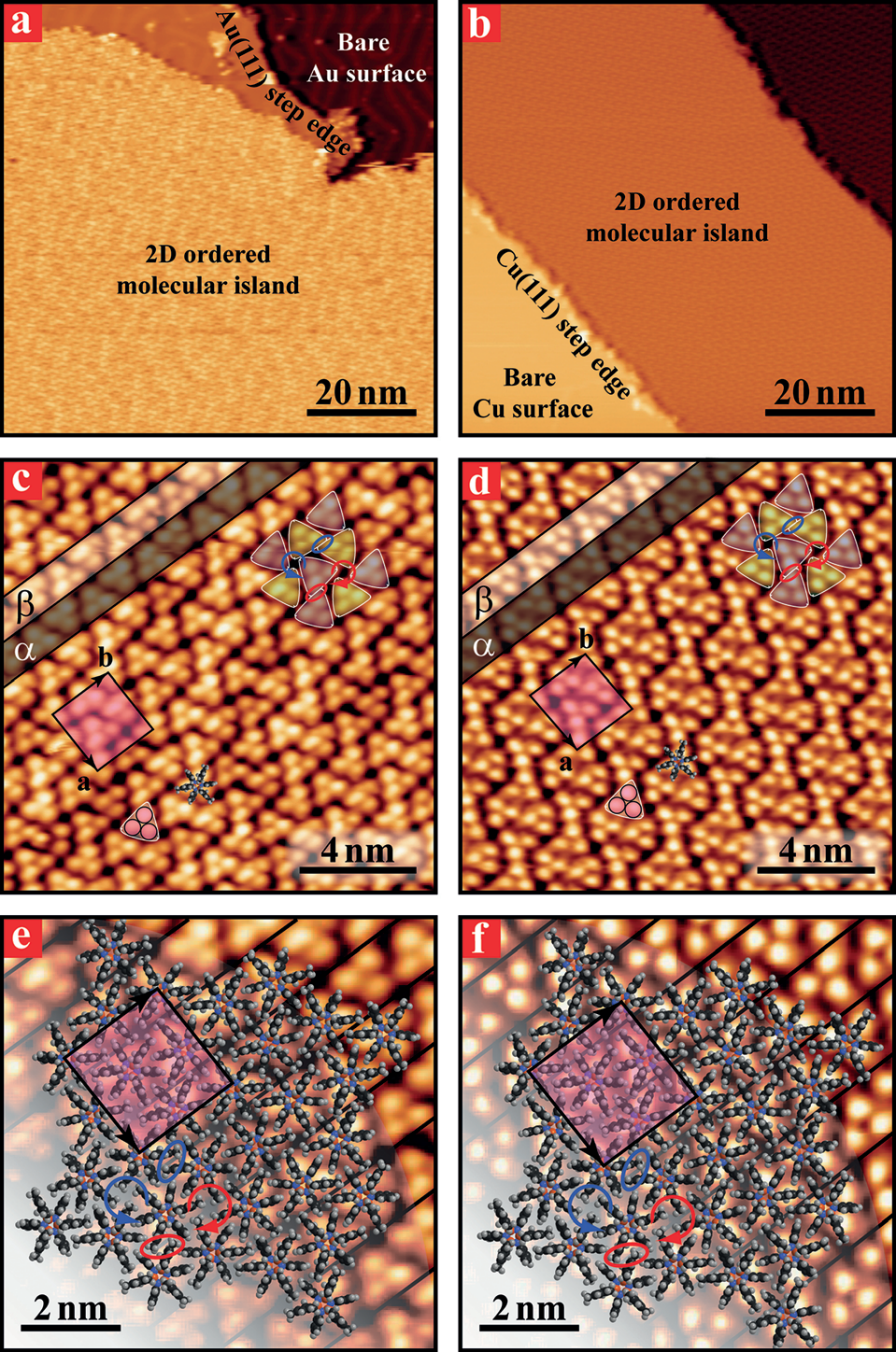
STM images of borazine 1 deposited on Au(111) (left panels) and Cu(111) (right panels) surfaces held at 300 K. The images were acquired at 77 K. Large views (a and b) of extended 2D supramolecular islands. Higher-resolution images (c and d) of parallel supramolecular stripes constituting the general assembly. The pink-shaded areas indicate the quasi-square unit cell. MD models (e and f) of the 2D molecular structure superimposed on the STM images.

At a submonolayer coverage, extended highly-ordered molecular islands of borazine **1** were observed on the terraces of Au(111) and Cu(111) surfaces (Figure 1 a and b). A close-up view of these islands shows that molecule **1** self-assembles into the very same close-packed network on both substrates, as it clearly appears from a comparison between Figure 1 c and d. This tiling pattern consists of rows formed by triangular features comprising three bright lobes. The distance of about 6 Å between the lobe centres within the triangles corresponds to that between the aromatic Ph or Mes rings in the molecular structure. Since the Mes substituents are bulkier than the Ph rings, one can assign a triangular set of lobes to the three Mes groups of a single molecule of **1**. The triangles alternate pointing upwards and downwards (green and purple outlines in Figure 1 c and d) within each row, and neighbouring rows are related by a C2 rotation (rows α and β highlighted in Figure 1 c and d).Two types of inter-row boundaries can be identified, in which pairs of molecules oriented in opposite directions (highlighted by either purple or green triangles) are arranged side-by-side (linkage is highlighted by red or blue ovals) across row borders. Due to this intermolecular arrangement, the molecules form a structure with a quasi-square unit cell defined by the following parameters ***a***=(22.9±0.6) Å, ***b***=(24.6±0.8) Å, and *θ*=(90±1.9) ° on both Au(111) and Cu(111) surfaces. A similar close-packed arrangement has been reported for six-fold hexaphenylbenzene hydrocarbons on these three-fold symmetry surfaces.^58^, ^59^,1

A modulation of the large and ordered molecular islands is visible on the Au(111) but not on Cu(111) surfaces (Figure 1 a and b). This corresponds to the characteristic herringbone reconstruction of Au(111) substrates, which influences the apparent STM height of the individual molecules (Figure 1 c and d). The fact that the reconstruction appears to be completely unaffected by the molecular adsorbates suggests the presence of feeble molecule–substrate interactions, most likely as a consequence of the surface-decoupling enforced by the bulky Mes substituents. This in turn suggests that short-range vdW attractive forces alone must govern the observed molecular packing arrangement.

We next performed MD calculations to rationalise the forces governing the distinctive organisation observed on the two substrates. The calculated equilibrium structures are in excellent agreement with the experimental images. A closer look at Figure 1 e and f reveals that both Mes and Ph groups of neighbouring molecules are deeply interdigitated, ensuing from vdW attractive forces. Specifically, a first type of vdW interaction occurs through offset parallel displacements of the Mes groups (red or blue ovals in Figure 1 e and f) belonging to those neighbouring molecules that are arranged side-by-side across the row borders (purple or green triangles in Figure 1 c and d). Another type of vdW interaction takes place between four adjacent molecules through perpendicular arrangement of their Mes groups, which can be described by clockwise or anticlockwise windmill motifs (see circular red or blue arrows in Figures 1 e and f). Notably, the supramolecular organisation observed in the STM images and re-obtained by MD simulation is the exact same on both Au(111) and Cu(111) surfaces, once more suggesting that the assembly behaviour of borazine **1** is not affected by the substrate chemical nature.

### Self-assembly of molecule 2 on Au(111) and Cu(111) surfaces

**Figure 2:**
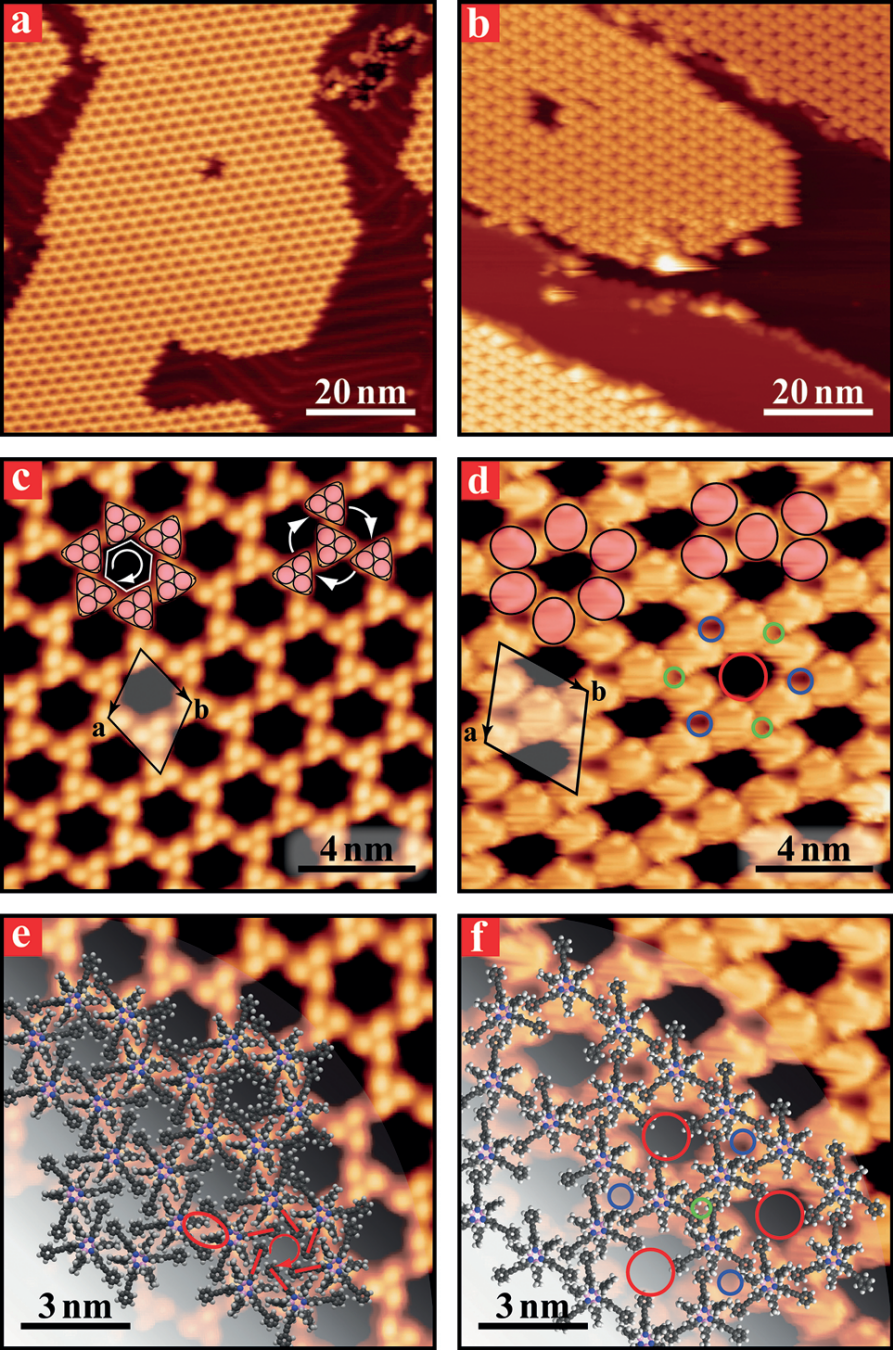
STM images of borazine 2 deposited at RT on Au(111) (left panels) and Cu(111) (right panels). The images were acquired at 77 K. Large views (a and b) of the extended 2D molecular islands. Higher resolution images (c and d) of the porous networks. MD structural models (e and f) of the molecular structure superimposed on the STM images. Images (c and e) show a clockwise chiral arrangement of the molecules decorating the pore on Au(111) surfaces (anticlockwise patterns are also observed).

Molecule **2** was deposited under similar conditions on Au(111) and Cu(111) surfaces held at room temperature (RT) at submonolayer coverage. Large scale STM images recorded at 77 K (Figure 2 a and b) show that, although the molecule assembles on both metals into porous networks forming large and well-ordered islands, the structure of the network differs between the two substrates (compare Figure 2 c and d).2

In particular, the deposition of molecule **2** on Au(111) results in a molecular honeycomb pattern built of triangular features comprising three bright lobes (Figure 2 c), very similar to those observed for molecule **1** (Figure 1 e and f). As for borazine **1**, a detailed analysis of the distance between adjacent lobes clearly shows that each can be assigned to the B-linked bulky Mes groups. Notably, the ethynyl-phenyl moieties are not visible in the STM images. This is essentially only due to a topographical effect since, as supported by MD simulations, the terminal Ph rings are likely to lie flat on the metal surface, about 5 Å below the Mes groups.

In this honeycomb structure, single molecules are in contact with three neighbours in a “propeller-like” arrangement with either clockwise or anticlockwise orientations (Figure 2 c and the Supporting Information, SI6 a and b). As a result, each molecule is surrounded by three large openings, each of which has hexagonal symmetry and is delimited by six molecules. The entire monolayer could be described as a hierarchical multilevel structure in which chiral clusters are wedged into each other yielding an overall chiral porous network with a rhombic unit cell with parameters **a**=**b**=(26.3±1.2) Å and *θ*=(60±4.5) ° (Figure 2 c).

The corresponding lowest energy configuration obtained from the MD simulations confirms the chiral ratchet-like arrangement of the phenyl-4-phenylethynyl groups within the pores conveying chirality to the entire network (Figure 2 e and the Supporting Information, SI7 a). Also in this case, the assembly is stabilised by short-range vdW interactions between offset parallel Mes groups of interdigitated neighbouring molecules (highlighted by the red contours in Figure 2 e).

A porous structure is also formed when molecule **2** is deposited on Cu(111) surfaces. The network assembly is built from what appear to be single, large, bright protrusions (pink filled ovals in Figure 2 d) with a size corresponding to an individual molecule. All molecules are linked with four neighbours in an X-shaped assembly geometry and separate two adjacent pores (red empty circles in Figure 2 d), each pore being surrounded by six molecules. Every molecule shares two further smaller pores of different size (blue and green empty circles in Figure 2 d), each surrounded by three molecules. The resulting hexagonal porous network has a rhombic unit cell with parameters **a**=**b**=(35.4±1.7) Å and *θ*=(60±4.8) °, which is significantly larger than that observed on Au(111).

MD simulations of this second type of porous network reveal that all the molecules adopt the same orientation with respect to the substrate and interact with their neighbours through different substituents. Consistent with the observations, three main types of voids characterise our calculated stable network structure: 1) The smallest pores (green circles in Figure 2 f), stemming from the interaction of strongly interdigitated (phenyl-4-phenylethynyl) groups of three neighbouring molecules; 2) The intermediate size pores (blue circles in Figure 2 f), also originating from the interaction of three molecules, this time through their (phenyl-4-phenylethynyl) and Mes groups; 3) The largest pores (red circles in Figure 2 f), deriving from alternating (phenyl-4-phenylethynyl) and Mes groups of six contiguous molecules.

**Figure 3:**
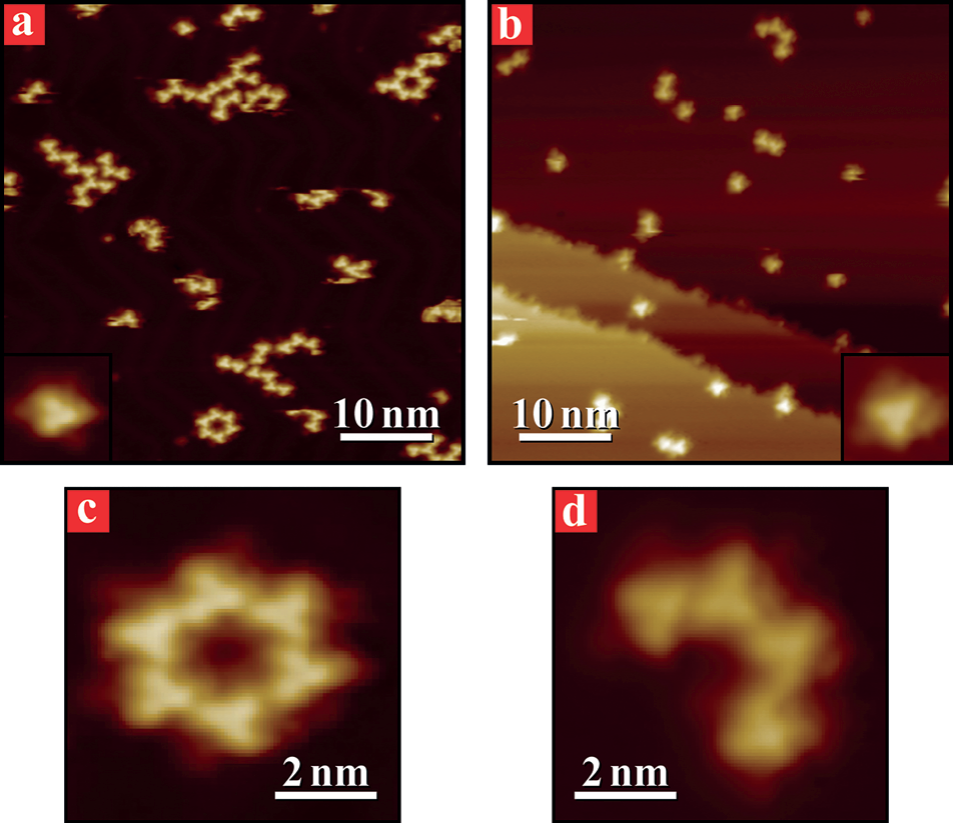
STM images of the assemblies formed by molecule 2 deposited on Au(111) (left panels) and Cu(111) (right panels) surfaces held at 140 K. Large-scale images (a and b) displaying molecular clusters of different sizes. Insets in (a and b) show individual molecules. High-resolution images of c) ring- and d) arc-like supramolecular architectures composed of six and four molecular units, respectively.

Low coverage deposition experiments of molecule **2** were also performed at 140 K on both substrates. These resulted in the formation of small clusters, composed from two to a few tens of molecules (Figure 3 a and b). All clusters are relatively stable over time and diffusing molecules were observed only occasionally. This stability is more pronounced on Cu(111) than on Au(111), suggesting stronger interactions for borazine **2** with the former substrate. On both surfaces, the isolated molecules are visualised as three bright lobes alternated by three dim spikes (insets of Figure 3 a and b). Whereas the former features clearly correspond to the Mes moieties, the latter can be assigned to the ethynyl-phenyl peripheries, proving that the molecules do not fragment during thermal sublimation. Larger clusters are organised into ring-like architectures, similar in size and structure to the ratchet motifs formed at RT on Au(111) surfaces. This observation is particularly interesting for Cu(111) surfaces (Figure 3 b and d), as these low-temperature clusters are denser and significantly different from those formed at RT on the same substrate (compare with Figure 2 f). However, the assemblies formed at 140 K appear to be metastable, as they irreversibly transform into the RT porous honeycomb in Figure 2 d and b when the sample is annealed at *T*≥300 K.

## Rationale

On both substrates, molecule **1** self-assembles in large and highly ordered molecular islands with an extremely low density of defects. The 2D suprastructure is characterised by an interdigitated packing of Ph substituents that, independent of the metal surface, is driven by intermolecular short-range vdW forces. All observations suggest extremely weak molecule–substrate interactions caused by an effective separation of the central molecular borazine core from the underlying metallic surface. The mutual steric hindrance between alternating Ph and Mes substituents in molecule **1**, force the substituents to adopt an almost perpendicular orientation with respect to the borazine core. As a consequence, molecule **1** physisorbs on both substrates, essentially not experiencing any in-plane modulation of the adsorption potential.

Coming now to molecule **2**, the interaction with the Au(111) surface is also relatively weak and the supramolecular assembly is again largely governed by intermolecular forces, with the molecular packing being driven by the vdW interdigitation of the aromatic rings. The resulting organisation can thus be seen as a natural extension of the molecular assembly of borazine **1**, in which the longer substituents space apart neighbouring molecules, yielding a porous molecular network.^60^, ^61^

**Figure 4:**
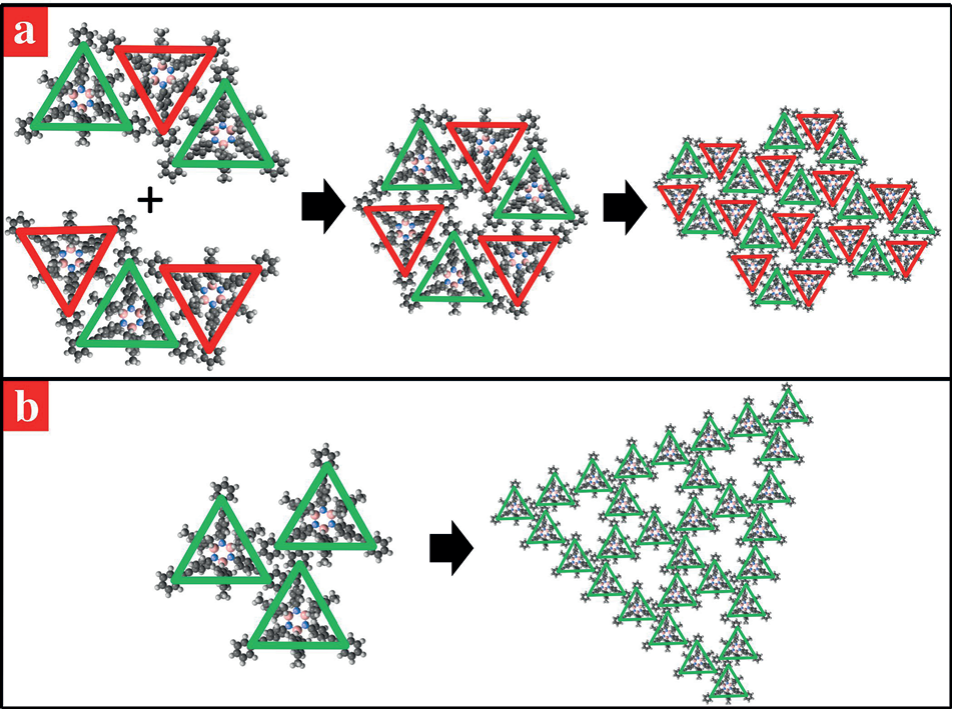
a) Schematic honeycomb structure of borazine 2 adsorbed on Au(111). The “anti-parallel” self-assembly mechanism ruling the formation of a single supramolecular hexagon is shown on the left. b) Schematic representation of the porous network on Cu(111) surfaces built upon the trimeric supramolecular cluster shown on the left.

A porous network is also formed by borazine **2** on Cu(111), but with a significantly different structure associated with a much lower degree of interdigitation. The relation between these two supramolecular arrangements can be visualised by considering the honeycomb assembly on Au(111) as being formed by molecules oriented in an alternating anti-parallel fashion (red and green triangles in Figure 4 a), which allows them to closely pack in a chiral hexagonal organisation. If every second molecule in this structure was rotated by 60 ° and the neighbouring molecules were allowed to rearrange to maximise vdW interactions (left-handed side in Figure 4 b), a new type of trimer would be formed that would constitute the basic unit of the porous hexagonal lattice observed on Cu(111) (right-handed side in Figure 4 b).4

It should be noticed that structure and size of the large pores in this lattice (red circles in Figure 2 d and f) are such that they could exactly accommodate one further borazine **2**. The molecular density in this theoretical assembly would then be 0.50 nm^−2^, that is, the same as the one associated with the honeycomb structure observed on Au(111). However, such a network of closed-packed interleaved trimeric units was never observed and all the STM measurements showed the porous networks in Figure 4 b, with a molecular density of 0.33 nm^−2^.

Evidently, the less dense arrangement of borazine **2** on Cu(111) implies an energy loss from vdW intermolecular interactions, which must be counterbalanced by a prevailing energetic gain. A possible explanation is provided by the attraction between the substrate and the terminal Ph rings in the phenyl-4-phenylethynyl moieties, which is expected to be stronger on the more reactive Cu(111) surface. When compared with the deposition of **2** on Au(111), the increased molecule–substrate interaction will enhance the relevance of the in-plane modulation of the adsorption potential, making some adsorption positions more favourable than others. We note that this is supported by the observation of two different molecular adsorption configurations for borazine **2** on Cu(111), which cause two different orientations for the porous network (the Supporting Information, Figure SI6 c and d).

**Figure 5:**
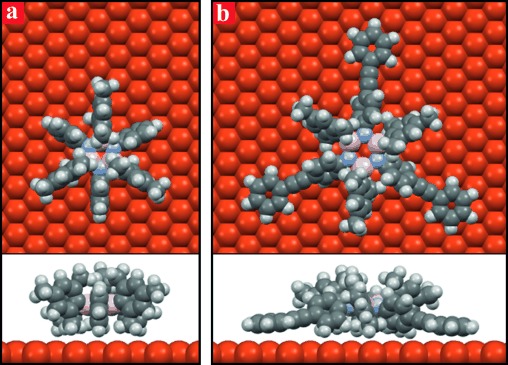
Calculated minimum energy adsorption configuration of borazines 2 (a) and 1 (b) on a Cu(111) surface. The steric hindrance between the Ph and Mes substituents results in the effective decoupling of the central borazine core from the surface for both molecules, whereas only the peripheral Ph rings of the phenylethynyl protrusions in borazine 1 experience a strong interaction with the metal surface.

As a consequence, the structure of the observed porous networks on Cu(111) can be seen as the result of a better epitaxial positioning of the Ph rings on the underlying substrate atomic lattice. This is different from the case of Au(111), in which the honeycomb network only derives from the vdW intermolecular forces because of the feebler interaction of **2** with this surface. At the same time, the stronger molecule–metal interaction on Cu(111) is also likely to induce a more pronounced deformation of molecule **2** (Figure 5). We speculate that a less closely packed assembly may also help moderate the molecular strain energy cost, therefore effectively increasing the relative stability of the observed porous structure. We notice that for both molecules **1** and **2**, the strong steric hindrance between the phenyl and mesityl substituents moieties imposes a lower limit on the adsorption height of the borazine ring, which our MD calculations evaluate as 5.4 Å for both molecules. This effective substrate decoupling of the molecular functional core is the result of the precisely targeted synthetic strategy we adopted.5

Finally, we note that the low coverage and temperature (140 K) development of small structures with a honeycomb symmetry on Cu(111) is only apparently in contradiction with the RT data. Indeed, low molecular density and high mobility should be expected to promote the formation of sparse dimers, trimers and tetramers to which the postulated cumulative surface-induced strain discussed above would not apply.

## Conclusion

We have studied for the first time the influence of lateral groups on the self-assembly of two borazine derivatives on metal substrates. By means of UHV-STM, various supramolecular structures have been observed for molecules **2** and **1**, all resulting from the interplay between intermolecular and molecule-substrate interactions. Molecule **1**, bearing Mes and Ph groups, forms identical close-packed networks on both Au(111) and Cu(111) surfaces, with a structure that is only governed by short-range attractive vdW interactions. In contrast, borazine derivative **2** arranges into porous motifs. Notably, the porosity of the networks was revealed to strongly depend on the nature of the metal substrate. Whereas more densely packed assemblies have been observed on Au(111), less compact networks of molecule **2** were imaged on Cu(111), in which stronger molecule–substrate interactions are most likely to be present.

Molecular dynamics simulations showed that this behaviour can be explained in terms of structural differences between the two borazines. In particular, the protruding phenyl-4-phenylethynyl substituents of molecule **2** act as intermolecular spacers, thus driving the formation of a porous network. Moreover, their flexibility allows a stronger interaction with the substrate which, in the case of the more reactive Cu(111) surface, causes a higher porosity.

It can be reasonably concluded that the central boron-nitride core does not exert any influence on the molecular ability to adsorb, self-assemble, or interact on different surfaces, thus proving the effectiveness of the synthetic strategy adopted to achieve molecule–substrate decoupling. As a consequence, it could be expected that combining similar types of all-carbon and BN-doped molecular isosteres in self-assembled architectures will contribute to the design of advanced molecular nanostructures for applications in electronic devices, in which an electrical decoupling from the metal substrates is required.

## Supporting Information

As a service to our authors and readers, this journal provides supporting information supplied by the authors. Such materials are peer reviewed and may be re-organized for online delivery, but are not copy-edited or typeset. Technical support issues arising from supporting information (other than missing files) should be addressed to the authors. miscellaneous_information
