# Supplementary material for: Self-Assembly of Decoupled Borazines on Metal Surfaces: The Role of the Peripheral Groups
Source: Chemistry. 2014 Jul 30;20(37):11856–62. doi: 10.1002/chem.201402839 (PMC4449113; doi:10.1002/chem.201402839)
Supplement: Supplementary file 1 — As a service to our authors and readers, this journal provides supporting information supplied by the authors. Such materials are peer reviewed and may be re-organized for online delivery, but are not copy-edited or typeset. Technical support issues arising from supporting information (other than missing files) should be addressed to the authors. miscellaneous_information [file chem0020-11856-sd1.pdf]

CHEMISTRY
A European Journal

Supporting Information

© Copyright Wiley-VCH Verlag GmbH & Co. KGaA, 69451 Weinheim, 2014

Self-Assembly of Decoupled Borazines on Metal Surfaces: The Role of the Peripheral Groups

Nataliya Kalashnyk,[a] Praveen Ganesh Nagaswaran,[c] Simon Kervyn,[c] Massimo Riello,[b] Ben Moreton,[a] Tim S. Jones,[a] Alessandro De Vita,*[b] Davide Bonifazi,*[c, d] and Giovanni Costantini*[a]

chem_201402839_sm_miscellaneous_information.pdf

- Supporting Information -

Experimental section

Synthesis and Characterization.

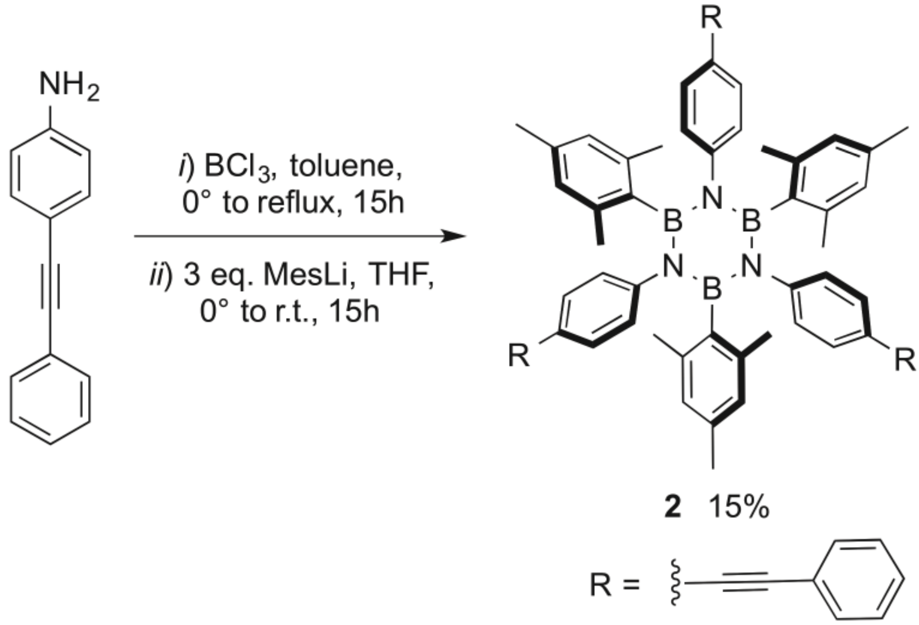

4-Ethynylphenyl aniline

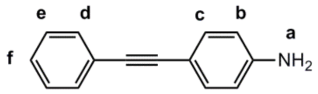

In a 250 mL two necked flask, 4-ethnylphenylnitrobenzene (8.5 mg, 0.038 mmol) was suspended in concentrated HCl (50 mL). The mixture was heated up to 60 °C under stirring to get a homogenous suspension. Then, tin powder (5 g, 42 mmol) was added slowly and the mixture was stirred at 60 °C for 6 h. After this period, a TLC showed complete conversion of the starting material and the solution was poured into 250 mL of ice (H_2O) and stirred to get a yellow suspension. Aqueous solution of NaOH (10%) was added to basify the suspension to pH 14. The

crude was extracted with EtOAc (3 × 100 mL), the combined organic layers were dried over $MgSO_4$, filtered and the solvents removed under reduced pressure. M.p.: 126-128 °C. Yield: 99%. ^1H NMR (400 MHz, C_6D_6, 25 °C) δ (ppm) 7.50-7.48 (m, 2H, H_d), 7.34-7.32 (m, 5H, H_c, H_e, H_f), 6.63 (d, J = 8 Hz, 2H, H_b), 3.83 (br, 2H, H_a); ^{13}C NMR (100 MHz, $CDCl_3$, 25 °C) δ (ppm) 146.6, 133.0, 131.4, 128.3, 127.7, 124.0, 114.8, 112.7, 90.2 (overlap of two signals). All other spectroscopic and analytical properties were identical to those reported in the literature (G. Adjabeng, T. Brenstrum, C. S. Frampton, A. J. Robertson, J. Hillhouse, J. McNulty, A. Capretta, *J. Org. Chem.* **2004**, *69*, 5082–5086).

B-Trimesityl-*N*-tri(4-ethynylphenylphenyl)borazine (**2**)

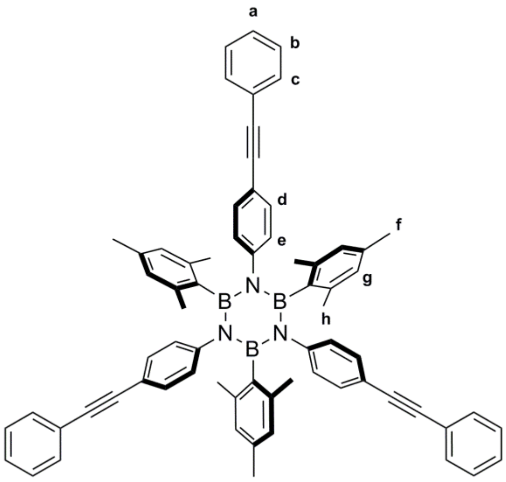

In a flame-dried 25 mL Schlenk flask, anhydrous 4-ethynylphenyl aniline (773 mg, 4 mmol) was diluted with 4 mL of anhydrous toluene and cooled to -5 °C (ice-salt bath). Then BCl_3 (4.8 mL, 4.8 mmol, 1M solution in toluene) was added dropwise yielding a white precipitate. The septum was changed for a dry condenser topped by a $CaCl_2$ tube. The reaction mixture was heated up to reflux overnight. After this period the flask was cooled down and subject to three freeze-to-thaw cycle to remove the HCl. In parallel, in a flame-dried 50mL Schlenk flask, MesBr (877 mg, 4.4 mmol) was diluted with 15 mL of anhydrous THF. The solution was cooled down to -78 °C and freshly titrated *n*-BuLi (3.4 mL, 4.8 mmol) was added dropwise. The flask was allowed to warm up to 0 °C for 1 h. The color changed from transparent to light yellow. The borazole was cannulated dropwise to the lithiate at 0 °C and allowed to react at r.t. After 17 h the reaction was quenched by H_2O. The aqueous layer was extracted with EtOAc (3 × 20 mL). The combined organic layers were dried over $MgSO_4$, filtered and the solvents removed under reduced pressure. The crude was purified by silica gel chromatography, CHX/CH_2Cl_2 (1:1) Rf: 0.5, to afford a white solid (195 mg, 15% yield). M.p.: >300 °C. ^{1}H NMR (400 MHz, C_6D_6, 25 °C) δ (ppm) 7.24-7.22 (m, 6H, H_d), 6.99 (d, J = 8 Hz, 6H, H_c), 6.95 (d, J = 8 Hz, 6H, H_b), 6.87-6.85 (m, 9H, H_a, H_e), 6.42 (s, 6H, H_g), 2.31 (s, 18H, H_h),

1.80 (s, 9H, H_f); ^{13}C NMR (100 MHz, C_6D_6, 25 °C) δ (ppm) 146.5, 137.2, 137.1, 131.4, 130.6, 128.1, 127.8, 127.4, 127.0, 123.7, 119.9, 89.7, 89.6, 23.0, 20.9 (the ^{13}C resonance corresponding to the carbon atom bonded to the boron atom is not observed due to the quadrupolar relaxation effect). ^{11}B NMR (128 MHz, $CDCl_3$, 25 °C) δ (ppm) 37.4 (br). Solid-state IR (KBr) ν (cm^{-1}) 2916 (C-H aromatic), 1510, 1356 (B-N) 1301, 836, 754. HRMS (MALDI, *m/z*): [$M^{+\cdot}$] calc. for $C_{69}H_{60}N_3B_3$, 963.5066; found, 963.5086.

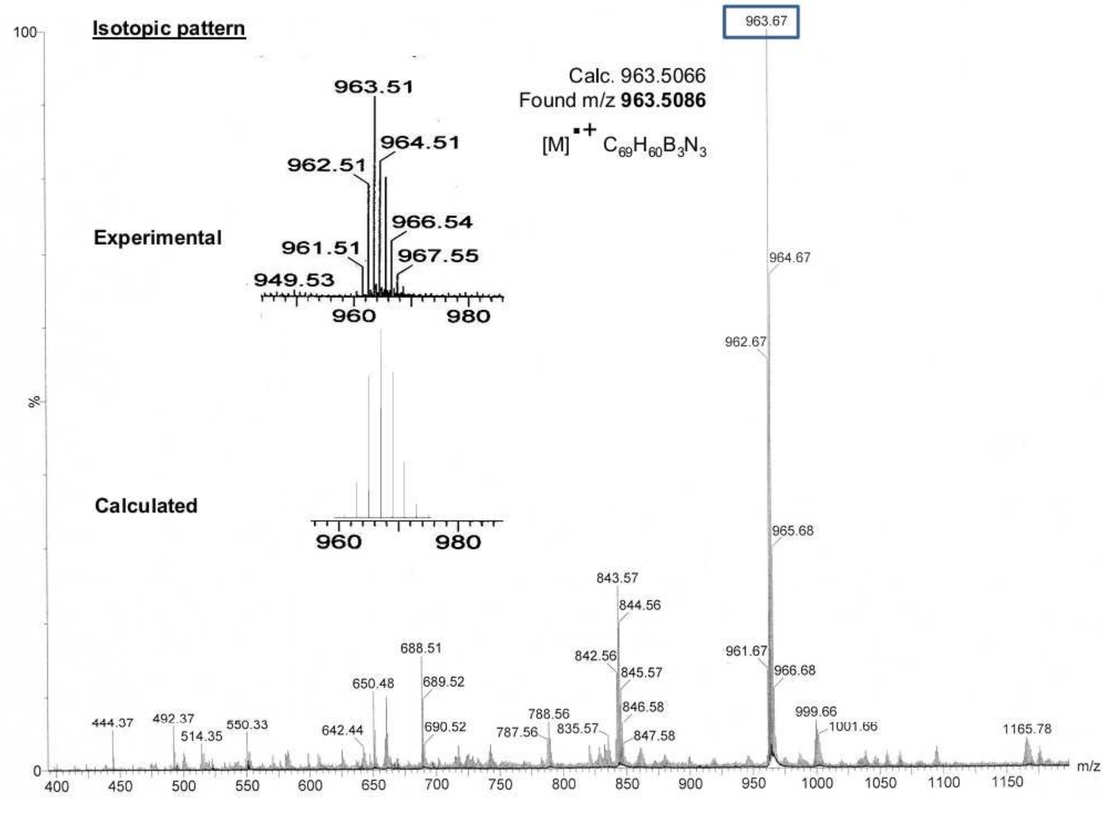

Figure SI1. Mass spectrum of borazine **2**, corresponding to the molecular formula: $C_{69}H_{60}B_3N_3$. MALDI-MS, inset: MALDI-HRMS, matrix: DCTB.

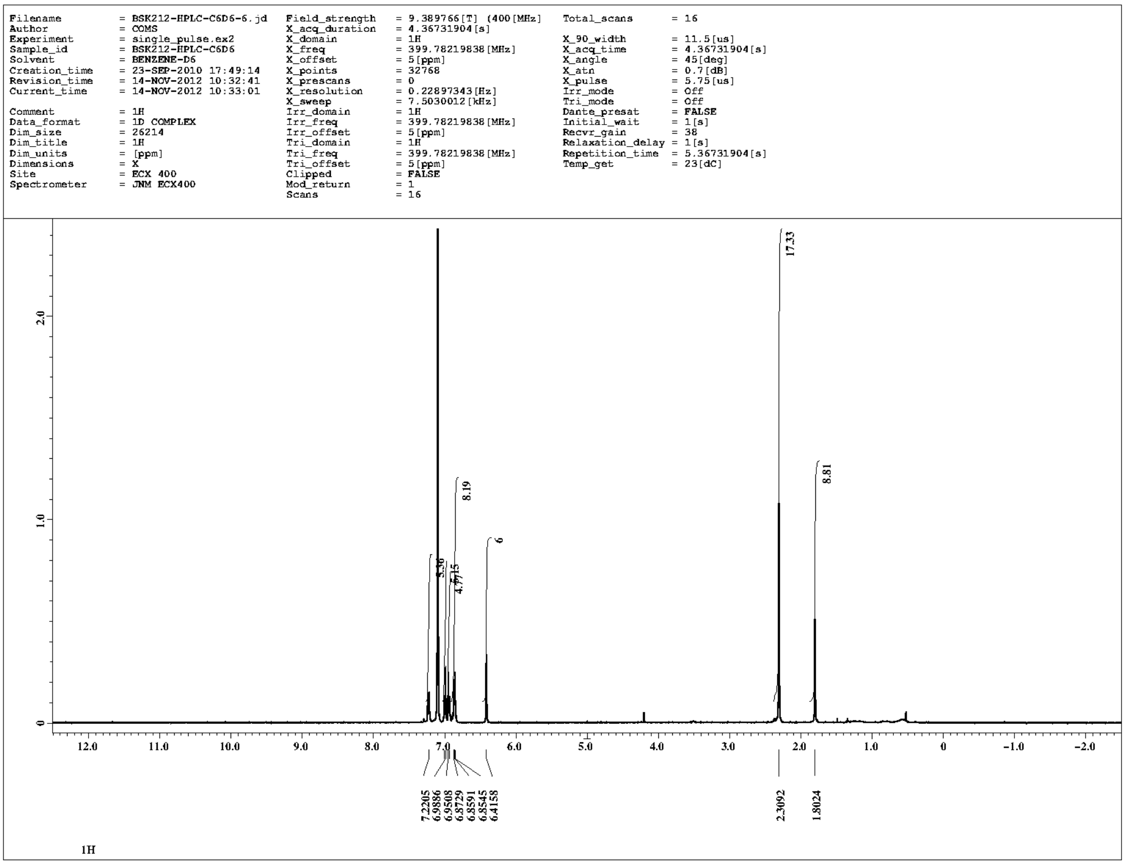

Figure SI2. ^{1}H NMR spectrum of borazine **2**.

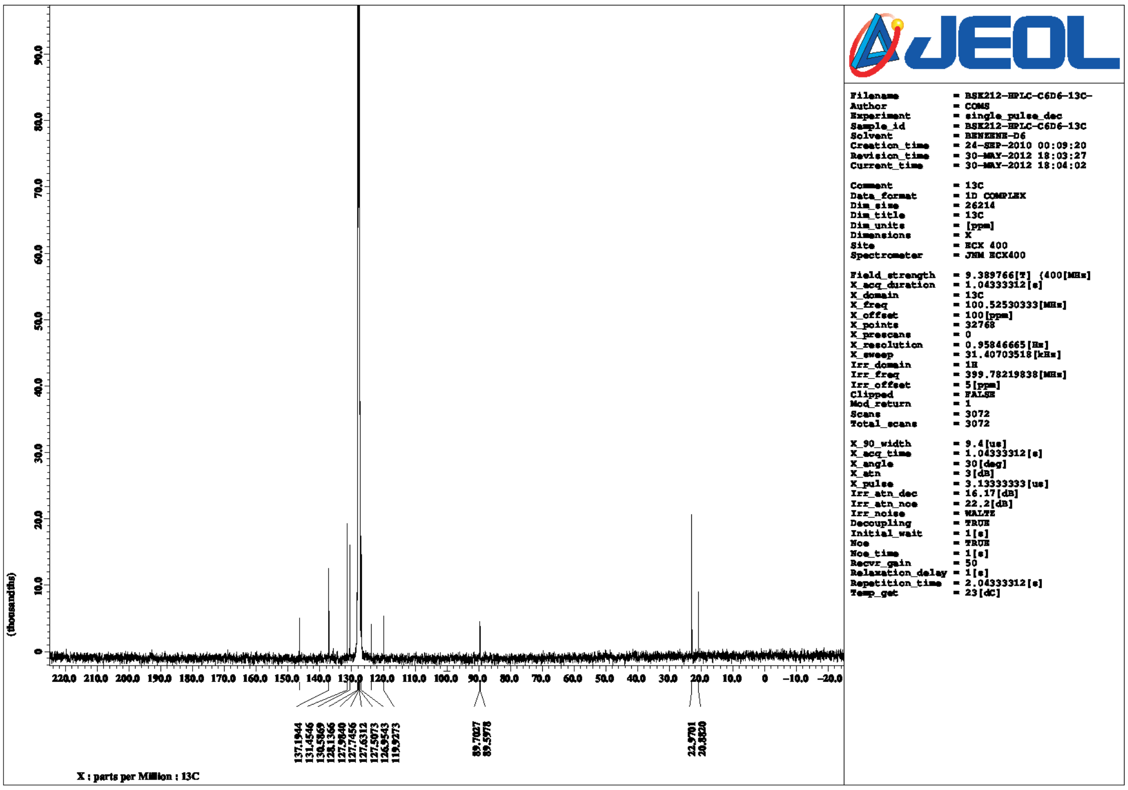

Figure SI3. ^{13}C-NMR spectrum of borazine **2**.

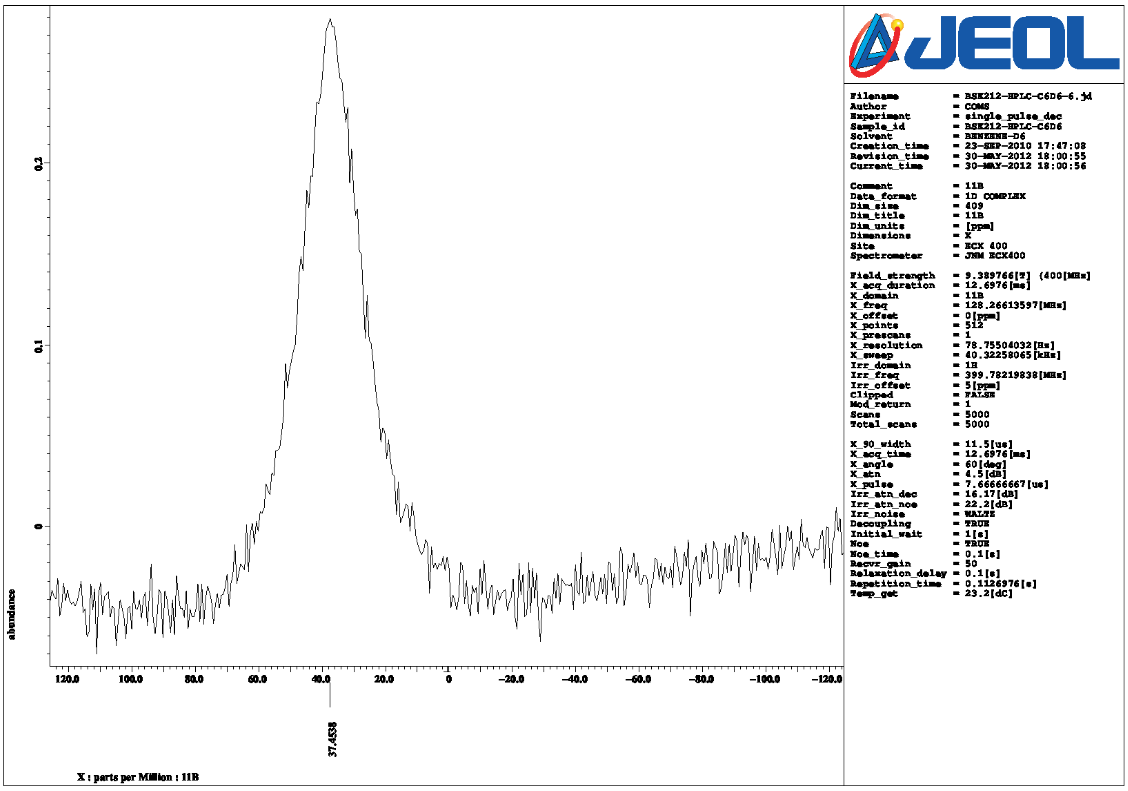

Figure SI4. ^{11}B-NMR spectrum of borazine **2**.

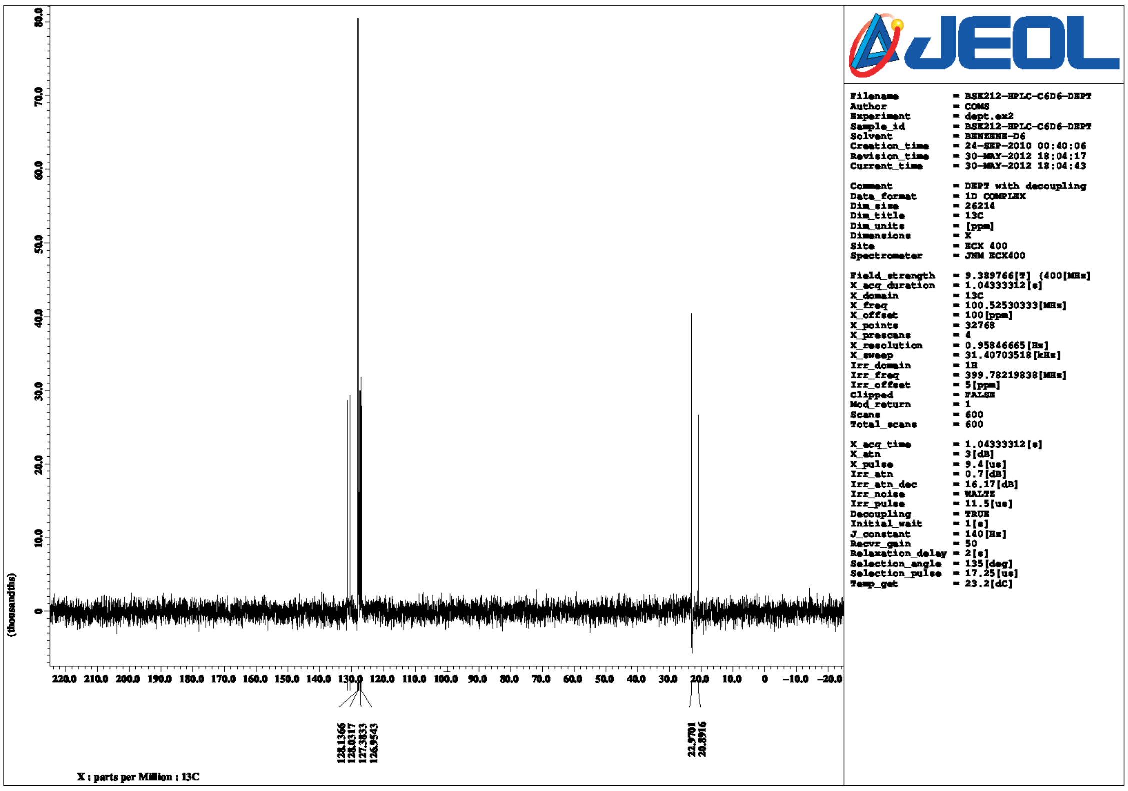

Figure SI5. DEPT-NMR spectrum of borazine **2**.

Scanning Tunnelling Microscopy experiments.

The Au(111) and Cu(111) substrates were cleaned by repeated cycles of Ar+ sputtering (1 keV, 15 μA/cm^2 for 15 min) followed by sample annealing (870 K, peak pressure of 2.10^{-9} mbar, for 5 min). The UHV transfer of molecules **1** and **2** onto the single crystal metal surfaces maintained either at RT or at 140 K was achieved by means of conventional thermal sublimation from a Knudsen cell. The cell temperatures were 553 K and 443 K for molecules **1** and **2**, respectively. The experimental data were acquired on a Createc LT-STM system at 77 K with a base pressure of 4.10^{-11} mbar. All STM images were typically recorded with a sample bias of 1.8 V and a tunneling current of 4.10^{-11} A and were subsequently processed using the WsXM software (I. Horcas, R. Fernández, J. M. Gómez-Rodríguez, J. Colchero, J. Gómez-Herrero, A. M. Baro, *Rev. Sci. Instrum.* **2007**, *78*, 013705).

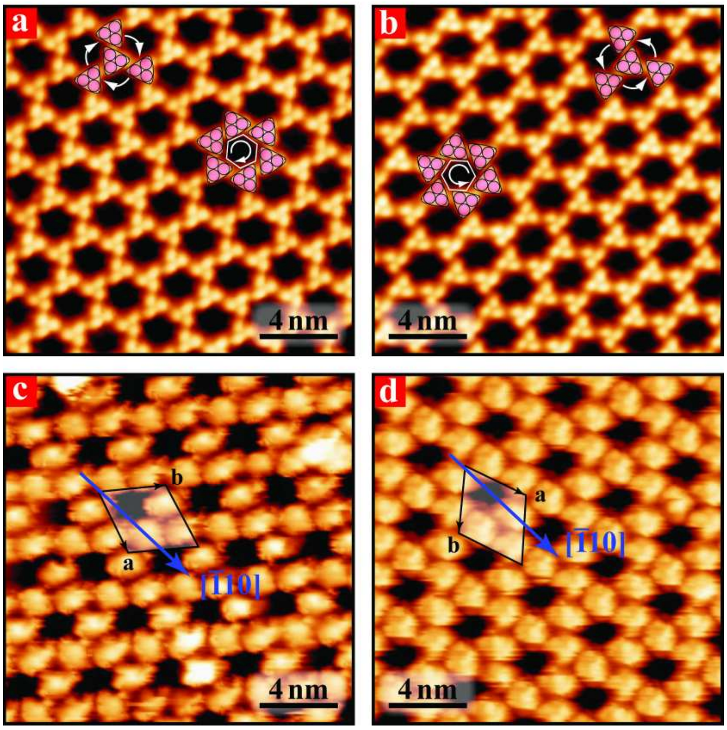

Figure SI6. Symmetry of the supramolecular structures formed by borazine **2** on Au(111), (a) and (b), and on Cu(111), (c) and (d). While on Au(111) the relative position of adjacent molecules causes organisational chirality, on Cu(111) the two different structures are obtained by reflection with respect to the $(\bar{1}\bar{1}1)$ plane and are most probably due to two equivalent molecular absorption configurations.

Molecular dynamics calculations.

Classical molecular dynamics simulations were carried out with the LAMMPS package (S. Plimpton, *J. Comp. Phys.* **1995**, *117*, 1-19), using the Universal Force-Field (UFF) to model the geometry and the intermolecular interactions of organic-substituted borazines **1** and **2** (A. K. Rappé *et al.*, *J. Am. Chem. Soc.* **1992**, *114*, 10024).

Interactions at the metal-organic interface were modelled accordingly to a semiempirical force-field fitted from desorption experiments and MP2 calculations of simple hydrocarbons adsorbed on Au(111) (F. Iori *et al.*, *J. Comput. Chem*. **2009**, *30*, 1456). This force-field parameterisation was assumed to remain valid also for the Cu(111) surface, since experimental adsorption energies of π-conjugated hydrocarbons – *e.g.*, ethylene and benzene – on Cu(111) (S. Lukas *et al., J. Chem. Phys.* **2001**, *114*, 10123) and Au(111) surfaces (S. Wetterer *et al*., *J. Phys. Chem. B* **1998**, *102*, 9266) are nearly matching. We note that recent quantum mechanical investigations of benzene adsorption on Au(111) and Cu(111) estimated the binding energy to be slightly higher on the latter substrate (by ~0.1 eV/molecule) (W. Liu *et al*., *New J. Phys.* **2013**, *15*, 053046). Obviously, this level of analysis is precluded for systems in the thousand atom size scale. However, the predicted higher adsorption energy of benzene on Cu(111)

is fully consistent with our experimental observations for borazine **2**.

Structural relaxation of the metal substrate was assumed to have negligible effect on the molecular self-assembly (T.E. Dirama *et al.*, *Langmuir* **2007**, *23*, 12208), hence, the position of the metal atoms was kept frozen by setting all their force components to zero.

B-metal and N-metal interactions were accounted for by a single set of Lennard-Jones parameters, which was enough to reach excellent agreement between the simulated structures and the experimental ones (cf. *e.g.*, Figures 1e,f and 2e,f in the main text). This approximation was further justified by the fact that, in borazines **1** and **2**, only the aryl substituents directly interact with the metal substrate (Figure 5, main text) or with neighbouring molecules (mainly via π-π stacking with similar aromatic groups) during the assembly (Figure SI6 and Figure 4, main text).

All calculations were carried out in the canonical ensemble, using a Langevin thermostat. Particle mesh Ewald was used to compute long-range electrostatic interactions based on a 1 Å mesh, and the cut-off for non-bonded terms was set to 10 Å.

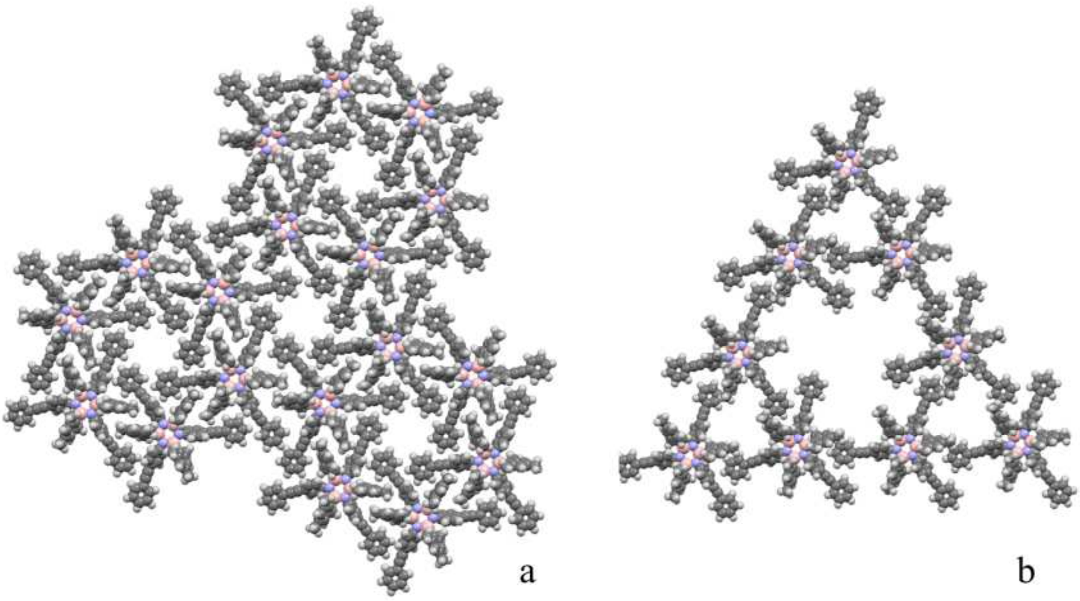

Figure SI7. Close-up view of the calculated models for the self-assembled structures of borazine **2** on Au(111) (a) and Cu(111) (b). The estimated surface densities are 0.50 molecules/nm^2 and 0.33 mols/nm^2 for (a) and (b), respectively.

We notice that the supramolecular arrangement calculated for borazine **2** on Cu(111) explains very well the experimentally observed triangular-shaped defects found in regions of the porous network where several molecules are missing (Figure SI8).

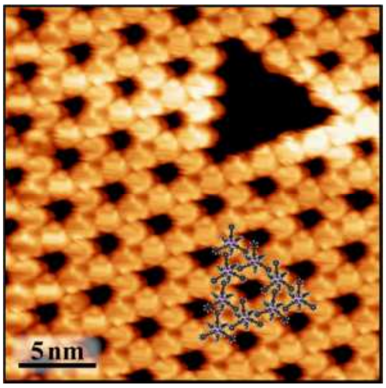

Figure SI8. Experimentally observed triangular-shaped defects found in the porous network formed at room temperature by borazine **2** on Cu(111).
